# Impact of differences in kidney function estimation by creatinine compared to cystatin C on clinical outcomes in patients with atrial fibrillation

**DOI:** 10.1093/ehjopen/oeag145

**Published:** 2026-09-22

**Authors:** Philipp Krisai, Mikko Mutti, Stefanie Aeschbacher, Michael Coslovsky, Tobias Reichlin, Nicolas Rodondi, Peter Ammann, Giorgio Moschovitis, Elia Rigamonti, Giulio Conte, Moa L Haller, Amelie Stoecklin, Leo H Bonati, Stefan Osswald, David Conen, Felix Mahfoud, Michael Kühne

**Affiliations:** Cardiology/Electrophysiology Division, Department of Medicine, University Hospital Basel, Petersgraben 4, 4031 Basel, Switzerland; Cardiovascular Research Institute Basel, University Hospital Basel, University of Basel, Spitalstrasse 2, 4056 Basel, Switzerland; Cardiology/Electrophysiology Division, Department of Medicine, University Hospital Basel, Petersgraben 4, 4031 Basel, Switzerland; Cardiovascular Research Institute Basel, University Hospital Basel, University of Basel, Spitalstrasse 2, 4056 Basel, Switzerland; Cardiology/Electrophysiology Division, Department of Medicine, University Hospital Basel, Petersgraben 4, 4031 Basel, Switzerland; Cardiovascular Research Institute Basel, University Hospital Basel, University of Basel, Spitalstrasse 2, 4056 Basel, Switzerland; Cardiovascular Research Institute Basel, University Hospital Basel, University of Basel, Spitalstrasse 2, 4056 Basel, Switzerland; Department of Clinical Research, University of Basel, University Hospital Basel, Spitalstrasse 8, 4056 Basel, Switzerland; Department of Cardiology, Inselspital, Bern University Hospital, University of Bern, Freiburgstrasse 20, 3010 Bern, Switzerland; Institute of Primary Health Care (BIHAM), University of Bern, Mittelstrasse 43, 3012 Bern, Switzerland; Department of General Internal Medicine, Inselspital, Bern University Hospital, University of Bern, Freiburgstrasse 20, 3010 Bern, Switzerland; Department of Cardiology, Kantonsspital St Gallen, Rorschacher Strasse 95, 9000 St Gallen, Switzerland; Division of Cardiology, Cardiocentro Ticino Institute, Ente Ospedaliero Cantonale, Via Tesserete 48, 6900 Lugano, Switzerland; Division of Cardiology, Cardiocentro Ticino Institute, Ente Ospedaliero Cantonale, Via Tesserete 48, 6900 Lugano, Switzerland; Division of Cardiology, Cardiocentro Ticino Institute, Ente Ospedaliero Cantonale, Via Tesserete 48, 6900 Lugano, Switzerland; Institute of Primary Health Care (BIHAM), University of Bern, Mittelstrasse 43, 3012 Bern, Switzerland; Department of General Internal Medicine, Inselspital, Bern University Hospital, University of Bern, Freiburgstrasse 20, 3010 Bern, Switzerland; Cardiology/Electrophysiology Division, Department of Medicine, University Hospital Basel, Petersgraben 4, 4031 Basel, Switzerland; Cardiovascular Research Institute Basel, University Hospital Basel, University of Basel, Spitalstrasse 2, 4056 Basel, Switzerland; Department of Neurology and Stroke Center, University Hospital Basel, University of Basel, Petersgraben 4, 4031 Basel, Switzerland; Research Department, Reha Rheinfelden, Salinenstrasse 98, 4310 Rheinfelden, Switzerland; Cardiology/Electrophysiology Division, Department of Medicine, University Hospital Basel, Petersgraben 4, 4031 Basel, Switzerland; Cardiovascular Research Institute Basel, University Hospital Basel, University of Basel, Spitalstrasse 2, 4056 Basel, Switzerland; Population Health Research Institute, McMaster University, 237 Barton Street East, Hamilton, ON L8L 2X2, Canada; Cardiology/Electrophysiology Division, Department of Medicine, University Hospital Basel, Petersgraben 4, 4031 Basel, Switzerland; Cardiovascular Research Institute Basel, University Hospital Basel, University of Basel, Spitalstrasse 2, 4056 Basel, Switzerland; Cardiology/Electrophysiology Division, Department of Medicine, University Hospital Basel, Petersgraben 4, 4031 Basel, Switzerland; Cardiovascular Research Institute Basel, University Hospital Basel, University of Basel, Spitalstrasse 2, 4056 Basel, Switzerland

**Keywords:** atrial fibrillation, cystatin C, creatinine, glomerular filtration rate, biomarker

## Abstract

**Aims:**

Differences between cystatin C-based and creatinine-based glomerular filtration rate estimations (eGFRdiff) have been associated with adverse outcomes. We aimed to investigate the prevalence of eGFRdiff and its association with clinical outcomes and brain lesions in patients with atrial fibrillation (AF).

**Methods and results:**

We analysed 3804 patients with AF enrolled in two prospective, multicentre cohort studies. The eGFRdiff was calculated by ‘cystatin C-based eGFR—creatinine-based eGFR’. Patients were stratified into three groups: eGFRdiff >10, −10 to 10, and <−10 mL/min/1.73 m². Associations with high-sensitivity troponin T (hs-TnT), NT-proBNP, major adverse cardiovascular (CV) events (stroke/systemic embolism, myocardial infarction and CV death), first heart failure (HF) hospitalization, and magnetic resonance imaging (MRI)-detected brain lesions were assessed. The mean age was 71 years, 28% were female, and 49% had paroxysmal AF. An eGFRdiff of >10, −10 to 10, and <−10 mL/min/1.73 m^2^ was present in 17.7%, 71.7%, and 10.6% of the patients, respectively. Incidence rates per 100 patient-years increased across these groups for major CV events (MACEs: 1.7, 3.9, 6.8) and HF hospitalization (1.2, 3.5, 6.1). Similarly, the prevalence of MRI-detected brain infarcts (28%, 39%, 42%) and moderate-to-severe white matter hyperintensities (38%, 55%, 63%) increased with greater eGFRdiff. The levels of hs-TnT and NT-proBNP were inversely correlated with eGFRdiff. In multivariable-adjusted linear models, lower eGFRdiff was associated with MACE (*P* = 0.006) and HF hospitalization (*P* = 0.015).

**Conclusion:**

A substantial proportion of patients with AF exhibit clinically relevant eGFRdiff. More negative eGFR difference was associated with elevated cardiac biomarkers, greater burden of ischaemic brain lesions, and increased risk of CV events and HF hospitalization.

**ClinicalTrials.gov Identifier:**

NCT02105844

## Graphical Abstract

**Graphical Abstract:**
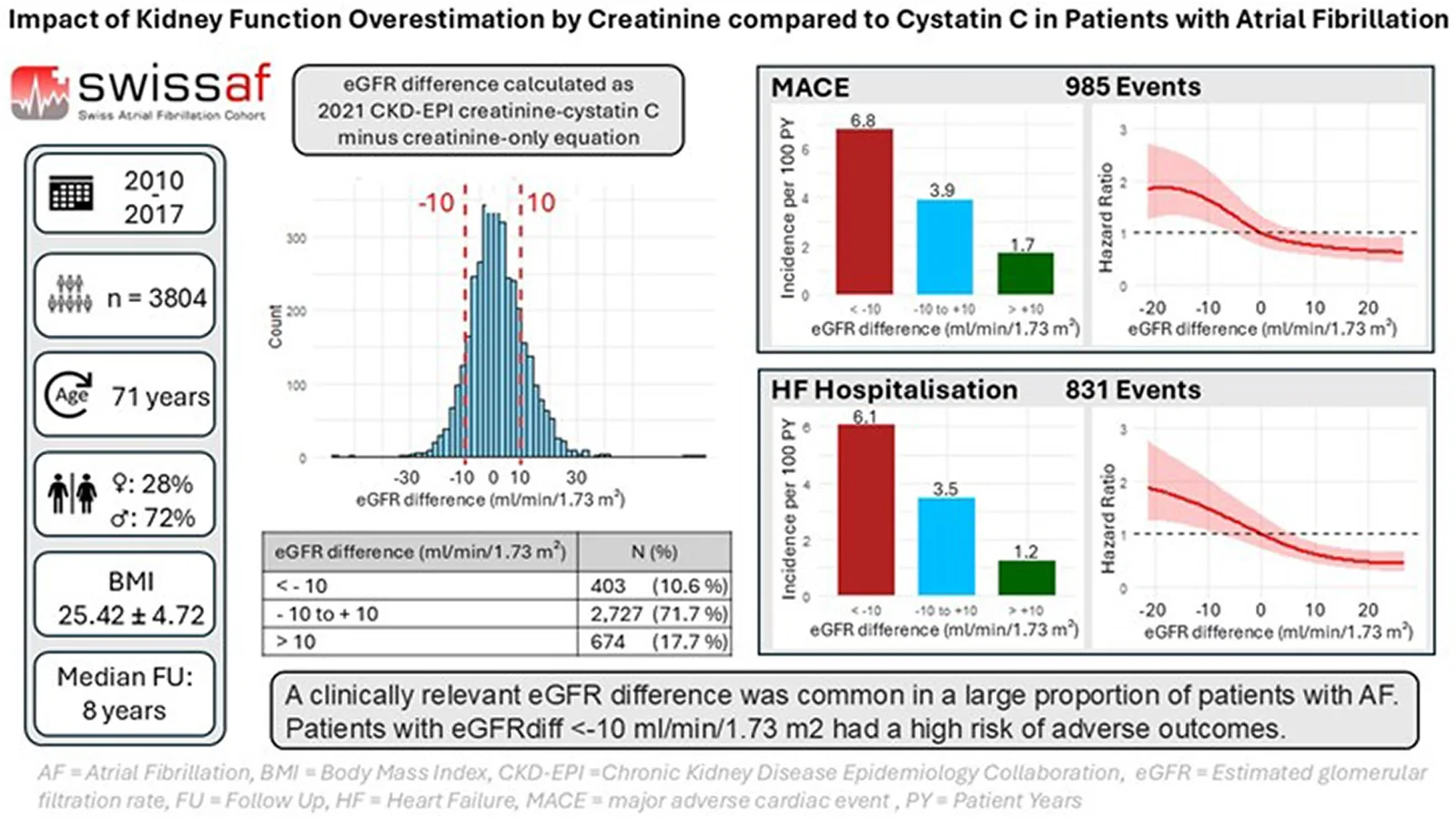

What’s new?

- Nearly 30% of patients with atrial fibrillation exhibit clinically relevant differences between creatinine-based and cystatin C-based glomerular filtration rate estimates (eGFRdiff).
- A more negative eGFRdiff identifies a high-risk phenotype with four-fold higher rates of major cardiovascular events and five-fold higher heart failure hospitalization.
- eGFR discrepancies correlate with elevated cardiac biomarkers and increased burden of MRI-detected brain lesions.
- Current reliance on creatinine-based estimates alone may lead to inappropriate anticoagulant dosing in a substantial proportion of atrial fibrillation patients.

## Introduction

About one-third of patients with atrial fibrillation (AF) have at least moderate renal impairment,^1^ which associates with increased risks of ischaemic events, heart failure (HF) hospitalization, and all-cause mortality.^2–4^ Kidney function in clinical practice is typically assessed using estimated glomerular filtration rate (eGFR) with creatinine as the standard biomarker. While creatinine-based eGFR may be sufficiently accurate in most clinical scenarios, it has several limitations such as dependency on nutritional status, body composition, and muscle mass, especially in elderly or comorbid patients.^5,6^ In contrast, cystatin C is less affected by these variables, and combined creatinine and cystatin C equations for eGFR are generally considered more accurate and robust.^7^

Intraindividual differences between creatinine-based and creatinine and cystatin C-based eGFR are common in chronic kidney disease (CKD).^8^ Moreover, eGFR differences have been associated with an increased risk of mortality in patients with CKD and HF.^8–10^ Specifically, when creatinine-based eGFR exceeds cystatin C-based estimates, this may reflect overestimation of renal function in advanced HF, with potential implications for drug dosing and prognostication.^9^ As HF remains the leading cause of death among patients with AF and considering the importance of appropriate dosing of direct oral anticoagulants (DOACs),^11,12^ discrepancies in eGFR estimation may have important clinical implications in the ever growing population.

In this extended analysis of a previous reported finding,^4^ we investigated the prevalence and extent of differences in eGFR between creatinine- vs. cystatin C-based equations, and their association with clinical outcomes and brain MRI-detected brain lesions in a large, multicentre cohort of patients with AF.

## Methods

### Study design

For this analysis, we combined data from two prospective, observational multicentre cohort studies, the Swiss Atrial Fibrillation Study (Swiss-AF) and the Basel Atrial Fibrillation Study (Beat-AF). Details of each study have been previously described.^13–15^ Briefly, both studies enrolled patients with documented AF and had similar study designs. In Swiss-AF, patients aged ≥65 years were enrolled, with a sub-population (10%) of patients <65 years to study health-related work absences, whereas there was no age restriction in Beat-AF. Main exclusion criteria in both studies were any acute illness in the last 4 weeks, non-sustained secondary forms of AF (e.g. due to surgery or infection), and inability to provide written informed consent. The screening and recruitment were performed on in- and outpatients in the participating hospitals and by contacting general practitioners. The studies were approved by the lead ethics committee, and all study centres received approval from the individual regional ethical committees.

Overall, the Swiss-AF study enrolled 2415 patients in 14 study centres between 2014 and 2017, and the Beat-AF study enrolled 1545 in 7 centres in Switzerland between 2010 and 2014. Of the 3960 enrolled patients, 156 had missing values for either biomarker or had no follow-up data, and were excluded from this analysis, which left 3804 individuals for the current analysis. The study flow is shown in Supplementary material online, *Figure S1*.

### Biomarkers and GFR estimation

Non-fasting venous blood samples were collected at enrolment. Blood samples were centrifuged, aliquoted in cryotubes, and stored in a biobank at −80°C. Biomarkers were measured after the enrolment of the last patient. Cystatin C and creatinine levels were measured on a Roche Cobas analyser (Roche, Switzerland). Nine biomarker values were below the lower limit of detection (0.4 mg/L for cystatin C, 5 μmol/L for creatinine) and were set to half of the biomarker specific cut-off. Two participants exceeded the upper limit of quantification for cystatin C, so the values were set to the specific lower limit cut-off of 6.8 mg/L.

We used the latest sex- and age-specific Chronic Kidney Disease Epidemiology Collaboration (CKD-EPI) formula to estimate a creatinine-based GFR (eGFRcrea) and a creatinine and cystatin C-based GFR (eGFRcrea-cys) (see Supplementary material online, *Table S1*).^7^ The difference in eGFR (eGFRdiff) was calculated as eGFRcrea-cys—eGFRcrea. Accordingly, higher eGFR by creatinine compared to creatinine and cystatin C results in a negative eGFRdiff and vice versa.

### Clinical outcomes

The two main clinical outcomes were major CV events (MACEs) and HF hospitalizations. The major CV event was defined as a composite of any stroke, systemic embolism, myocardial infarction, and cardiovascular (CV) death. Secondary outcomes included the individual components of MACE, all-cause death, and major bleeding. Detailed outcome definitions are provided in the Supplementary material. All clinical outcomes were assessed yearly by telephone, clinical visit and/or chart review, and were blindly validated by two independent clinicians. In case of disagreement, a third reviewer was consulted.

### Brain magnetic resonance imaging

Brain magnetic resonance imaging (MRI) at study enrolment was available for 1722 patients from the Swiss-AF study. 693 patients from the Swiss-AF study did not undergo MRI, mainly due to cardiac devices or claustrophobia. No brain MRI was performed in Beat-AF. Details of the standardized brain MRI protocols were previously published.^16,17^ Ischaemic brain infarcts were classified as large noncortical or cortical infarcts (LNCCIs) or small noncortical infarcts (SNCIs). Noncortical infarcts >20 mm were defined as large noncortical infarcts, while cortical infarcts were defined as hyperintense lesions on FLAIR involving the cortex, regardless of the size. The SNCIs were defined as lesions measuring ≤20 mm on FLAIR without involving the cortex. White matter hyperintensities (WMHs) were graded according to the Fazekas scale. A score ≥2 was defined as at least moderate disease and considered relevant for this analysis.^18^ Microbleeds were identified as nodular, strongly hypointense lesions on T2*weighted or susceptibility-weighted imaging.

### Other study variables

Patient demographics, past and current medical history, lifestyle factors, and medication were assessed by trained study personnel using standardized case report forms. At baseline, blood pressure was measured three times. The mean of all measurements was calculated and used for this analysis. A 12- or 16-lead electrocardiogram was performed, and the rhythm was defined as sinus rhythm, AF, or any other rhythm. The AF type classification was performed using the 2010 Guidelines of the Task Force for the Management of Atrial Fibrillation of the European Society of Cardiology.^19^

### Statistical analysis

Baseline characteristics and MRI data are shown overall and stratified by three eGFRdiff categories: <−10, −10 to 10, and >10 mL/min/1.73 m^2^. These categories were chosen based on prior evidence in patients with HF.^9^ Continuous data are presented as mean ± standard deviation or median (interquartile range) and compared with one-way ANOVA or Kruskal–Wallis tests, in case strongly skewed. Categorical data are presented as numbers (percentages) and compared with Pearson’s Chi-square tests.

The distribution of eGFRdiff was plotted for the whole study population. Cystatin C, creatinine, and their respective eGFR are plotted against NT-proBNP and hs-TnT levels. Incidence rates of the main and secondary outcomes were calculated as the number of events per 100 patient-years. Similarly, the Kaplan–Meier curves for each main outcome are stratified by the three predefined eGFRdiff groups and group comparisons were performed using the log-rank test. In addition, we used eGFRdiff as a continuous and categorial variable in two different Cox proportional hazards models to estimate hazard ratios of clinical outcomes. We used eGFRdiff as a categorical variable using the predefined three-group classification. Categorial models were constructed with the eGFRdiff group −10 to 10 mL/min/1.73 m^2^ as the reference group. For continuous models, we used natural cubic splines as the associations of eGFRdiff and the main outcomes were not linear. First, we used age- and sex-adjusted models. Second, we additionally adjusted for diabetes, arterial blood pressure, history of stroke/TIA, and smoking status. The proportional hazards assumption was tested using the Schoenfeld residuals. Additionally, interaction terms between eGFR difference (modelled continuously and categorically) and HF status at baseline were included in Cox proportional hazards models for the main outcomes to assess potential effect modification. As we found no significant interaction (*P* for interaction 0.45 for MACE, 0.61 for HF hospitalization), we did not perform stratified analyses in patients with and without HF at baseline. For the two main clinical outcomes, we estimated the sensitivity and specificity of our pre-specified eGFRdiff cut-offs (−10 and 10 mL/min/1.73 m^2^, respectively) and calculated an optimal cut-off using the Youden index for different follow-up periods. We additionally performed time-dependent calculations of the area under the curve (AUC) at 10 years for MACE and HF hospitalization using inverse probability of censoring weights (IPCW) for eGFRdiff, eGFRcrea vs. eGFRcrea-cysc and investigated the increase in AUC by adding eGFRdiff to eGFRcrea or eGFRcrea-cys, respectively, in the multi-adjusted models.

All analyses were performed using R (version 4.4.2), and a two-sided *P*-value of 0.05 was considered statistically significant. No correction for multiple testing was done.

## Results

### Baseline characteristics

Baseline characteristics are shown in *Table 1*. The mean age of the population was 71 ± 10 years, 2743 (72%) were male. The mean CHA_2_DS_2_-VASc score was 3.2 ± 1.7 and 1862 (49%) patients had paroxysmal AF. Baseline characteristics stratified by study cohort are shown in Supplementary material online, *Table S1*.

**Table 1:** Baseline characteristics stratified by the combined eGFR categories

	Overall	eGFR difference (mL/min/1.73 m^2^)			*P*-value
		<−10	−10 to 10	>10	
*N*	3804	403	2727	674	
Age (years)	71 (10)	74 (9)	73 (9)	64 (10)	<0.001
Sex (male)	2743 (72%)	283 (70%)	1914 (70%)	546 (81%)	<0.001
BMI (kg/m^2^)	27.4 (4.7)	27.9 (5.5)	27.4 (4.7)	27.1 (4.2)	0.481
Systolic BP (mmHg)	134 (19)	133 (21)	134 (19)	134 (17)	0.577
Diastolic BP (mmHg)	78 (12)	76 (12)	77 (12)	80 (11)	<0.001
Heart rate (bpm)	66 (58; 78)	70 (61; 80)	66 (59; 78)	64 (57; 75)	<0.001
Active smoking	298 (7.9%)	46 (11%)	198 (7.3%)	54 (8.0%)	0.015
CHA_2_DS_2_-VASc Score	3.2 (1.7)	3.8 (1.6)	3.4 (1.7)	2.1 (1.6)	<0.001
AF type					<0.001
Paroxysmal	1862 (49%)	155 (39%)	1311 (48%)	396 (59%)	
Non-paroxysmal	1939 (51%)	247 (61%)	1414 (52%)	278 (41%)	
Rhythm at baseline					<0.001
SR	1871 (50%)	131 (33%)	1288 (47%)	452 (67%)	
AF	1466 (39%)	214 (54%)	1082 (40%)	170 (25%)	
Other	441 (11%)	50 (13%)	343 (13%)	48 (7%)	
Medical history					
Prior pulmonary vein isolation	807 (21%)	54 (13%)	545 (20%)	208 (31%)	<0.001
Coronary artery disease	1017 (27%)	119 (30%)	792 (29%)	106 (16%)	<0.001
Prior stroke/TIA	647 (17%)	87 (22%)	474 (17%)	86 (13%)	<0.001
Hypertension	2626 (69%)	303 (75%)	1946 (71%)	377 (56%)	<0.001
Heart failure	903 (24%)	135 (33%)	685 (25%)	83 (12%)	<0.001
Diabetes	614 (16%)	86 (21%)	460 (17%)	68 (10%)	<0.001
Prior myocardial infarction	562 (15%)	58 (14%)	448 (16%)	56 (8.3%)	<0.001
Prior major/clinically relevant non-major bleeding	501 (13%)	72 (18%)	387 (14%)	42 (6.2%)	<0.001
Prior systemic embolism	190 (5.0%)	30 (7.4%)	135 (5.0%)	25 (3.7%)	0.024
Biomarker and eGFR					
Creatinine (mg/dL)	1.10 (0.94; 1.31)	0.98 (0.86; 1.13)	1.13 (0.96; 1.37)	1.05 (0.93; 1.19)	<0.001
Cystatin C (mg/dL)	1.12 (0.93; 1.41)	1.41 (1.23; 1.70)	1.17 (1.00; 1.44)	0.80 (0.72; 0.89)	<0.001
eGFR_crea_ (mL/min/1.73 m^2^)	66.88 (53.17; 80.87)	73.05 (61.65; 85.74)	63.02 (49.35; 77.45)	76.11 (64.04; 87.10)	<0.001
eGFR_crea-cys_ (mL/min/1.73 m^2^)	67.98 (51.21; 84.66)	58.22 (46.78; 70.38)	63.73 (47.96; 78.55)	93.49 (81.11; 104.27)	<0.001
eGFR_diff_ (mL/min/1.73 m^2^)	0.73 (−4.90; 7.42)	−13.50 (−16.73; −11.57)	−0.06 (−3.96; 4.03)	14.89 (12.16; 19.09)	<0.001
Hs-TnT (ng/L)	12.97 (8.42; 21.15)	18.40 (12.32; 33.01)	13.79 (9.13; 22.27)	8.37 (6.06; 11.76)	<0.001
NT-proBNP (ng/L)	571 (176; 1;343)	1;086 (433; 1;871)	650 (218; 1;448)	178 (80; 558)	<0.001
Medication					
β Blockers	2617 (69%)	295 (73%)	1910 (70%)	412 (61%)	<0.001
ACE inhibitors	1099 (29%)	124 (31%)	829 (30%)	146 (22%)	<0.001
Calcium antagonist	759 (20%)	567 (21%)	91 (23%)	101 (15%)	0.001
ARB	1153 (30%)	863 (32%)	133 (33%)	157 (23%)	<0.001
Digoxin	158 (4%)	106 (4%)	35 (9%)	17 (3%)	<0.001
Diuretics	1597 (42%)	1232 (45%)	244 (61%)	121 (18%)	<0.001
DOAK	1342 (35%)	160 (40%)	1010 (37%)	172 (26%)	<0.001
Vitamin K antagonists	1856 (49%)	192 (48%)	1321 (48%)	343 (51%)	0.448
Antiplatelet therapy	820 (22%)	101 (25%)	593 (22%)	126 (19%)	0.045
AAD, Class Ic and III	891 (23%)	47 (12%)	612 (22%)	232 (34%)	<0.001

Data are presented as mean (standard deviation), median (interquartile range), or numbers (percentage).

AAD, antiarrhythmic drugs; ACE, angiotensin converting enzyme; AF, Atrial fibrillation; ARB, angiotensin-II receptor blocker; BMI, body mass index; BP, blood pressure; DOAC, direct oral anticoagulant; ECG, electrocardiogram; eGFR, estimated glomerular filtration rate; Hs-TNT, high-sensitivity troponin T; SR, sinus rhythm; TIA, transient ischaemic attack; VKA, vitamin K antagonist.

The eGFRdiff showed a bell-shaped distribution (*Figure 1*) and the median eGFRdiff was 0.73 (−4.90; 7.42) mL/min/1.73 m^2^. Among the 3804 patients included, 403 (10.6%) had an eGFRdiff <−10 mL/min/1.73 m^2^, 2727 (71.6%) had an eGFRdiff from −10 to 10 mL/min/1.73 m^2^ and 674 (17.7%) had an eGFRdiff >10 mL/min/1.73 m^2^. Patients in the lowest eGFRdiff group were older, more often female, had a higher CHA_2_DS_2_-VASc score, more often non-paroxysmal AF and a greater burden of comorbidities (*Table 1*).

**Figure 1:**
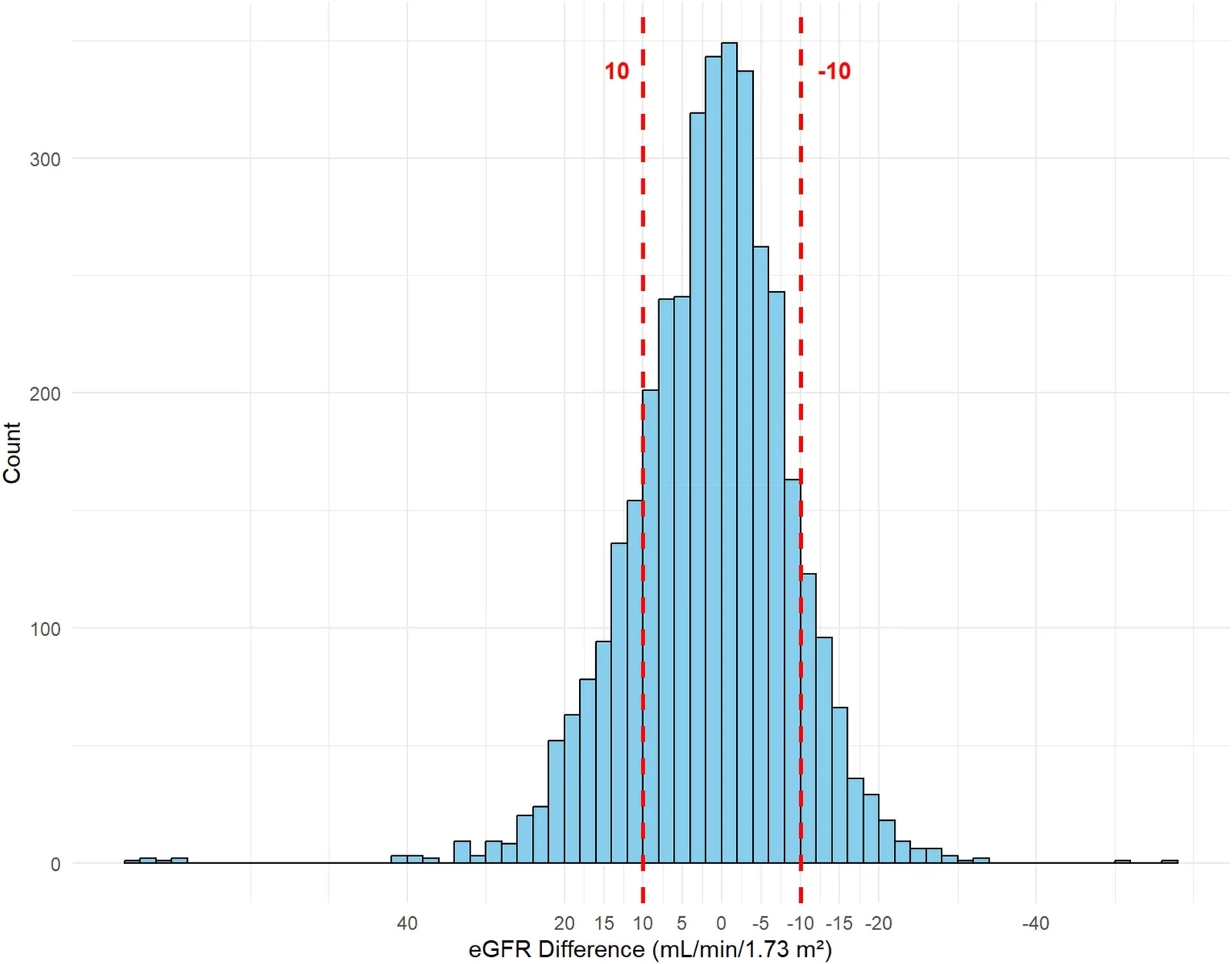
Distribution of estimated glomerular filtration rate differences.

### MRI detected brain lesions

Results of 1722 patients with available brain MRI are shown in *Table 2* and *Figure 2*. Overall, 38% of patients had evidence of any SNCI and/or LNCCI, 53% had at least moderate WMH (Fazekas ≥2) and 22% had microbleeds. Across the three eGFR groups the brain lesion prevalence increased from 28% to 42% (*P* = 0.006) for SNCI and/or LNCCI and from 38% to 63% (*P* < 0.001) for WMH (Fazekas ≥2). Prevalent microbleeds were not different between groups (18%, 22% and 25%; *P* = 0.2).

**Figure 2:**
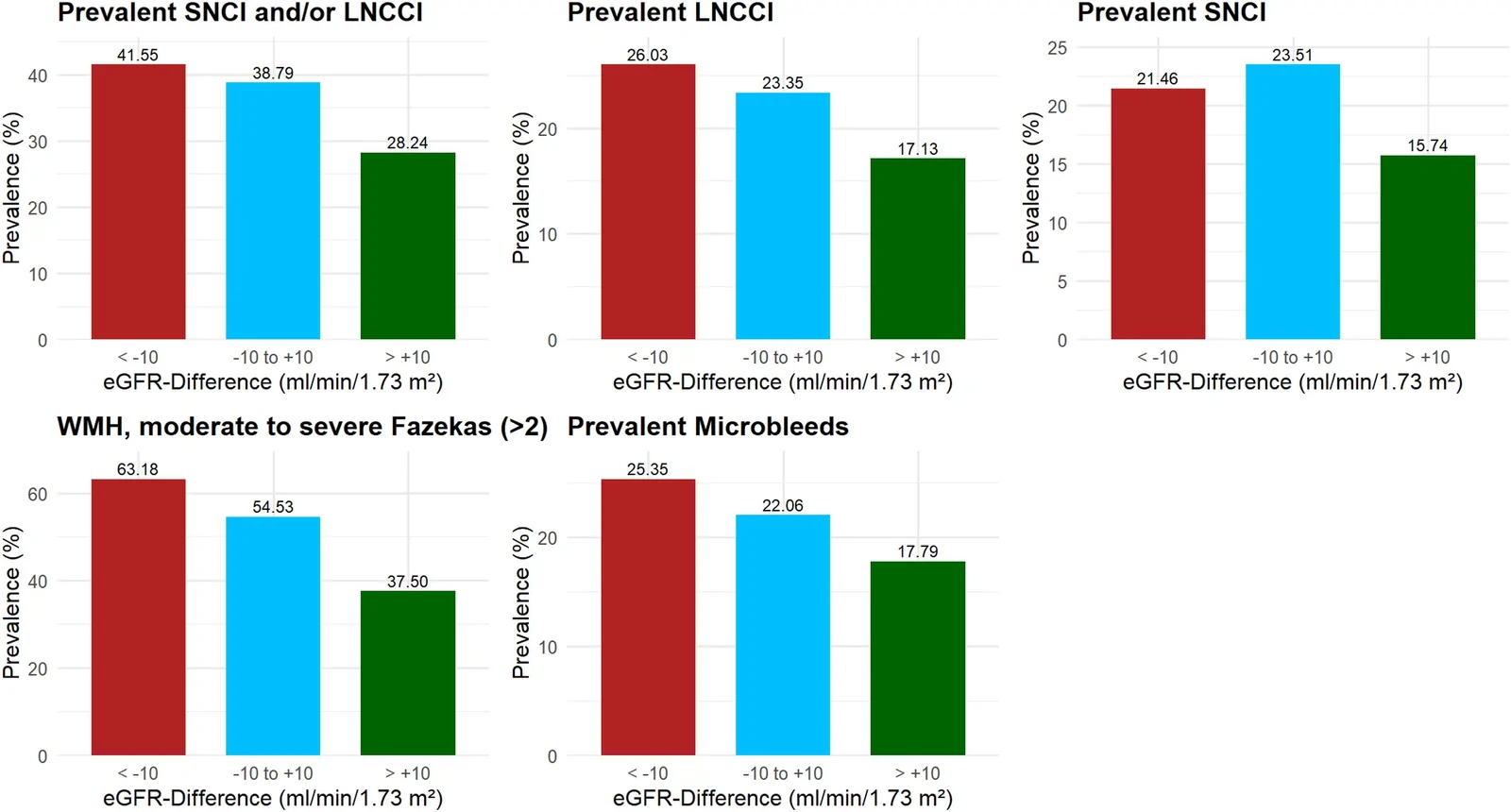
Prevalence of brain magnetic resonance imaging lesions by estimated glomerular filtration rate difference groups. eGFRdiff = eGFRcrea-cys – eGFRcrea. LNCCI, large non-cortical and cortical infarct; SNCI, small non-cortical infarct; WMH, white matter hyperintensity.

**Table 2:** Prevalence of brain lesions overall and according to estimated GFR difference group

	Overall	eGFR difference (mL/min/1.73 m^2^)			*P*-value
		<−10	−10 to 10	>10	
***N***	1722	220	1286	216	
**SNCI and/or LNCCI**	647 (38%)	91 (42%)	495 (39%)	61 (28%)	0.006
**LNCCI**	392 (23%)	57 (26%)	298 (23%)	37 (17%)	0.066
**SNCI**	381 (22%)	47 (21%)	300 (24%)	34 (16%)	0.038
**WMH (Fazekas ≥2)**	918 (53%)	139 (63%)	698 (55%)	81 (38%)	<0.001
**Microbleeds**	365 (22%)	54 (25%)	274 (22%)	37 (18%)	0.17

Values are presented as count (percentage), *P*-values were calculated Pearson’s chi-square tests.

eGFRdiff = eGFRcrea-cys – eGFRcrea.

LNCCI, large non-cortical and cortical infarct; SNCI, small non-cortical infarct; WMH, white matter hyperintensity.

### eGFR difference and cardiac biomarkers

Biomarker levels of hs-TnT and NT-proBNP increased continuously across decreasing eGFRdiff (*Figure 3*). Across the three eGFR groups hs-TnT levels were 8.4 (6.1; 11.8), 13.8 (9.1; 22.3) and 18.4 (12.3; 33.0) ng/L and NT-proBNP levels were 178 (90; 558), 650 (218; 1448) and 1086 (433; 1871) ng/L, respectively (*Table 1*). The relation of creatinine and cystatin C with these two biomarkers is shown in Supplementary material online, *Figures S2* and *S3*.

**Figure 3:**
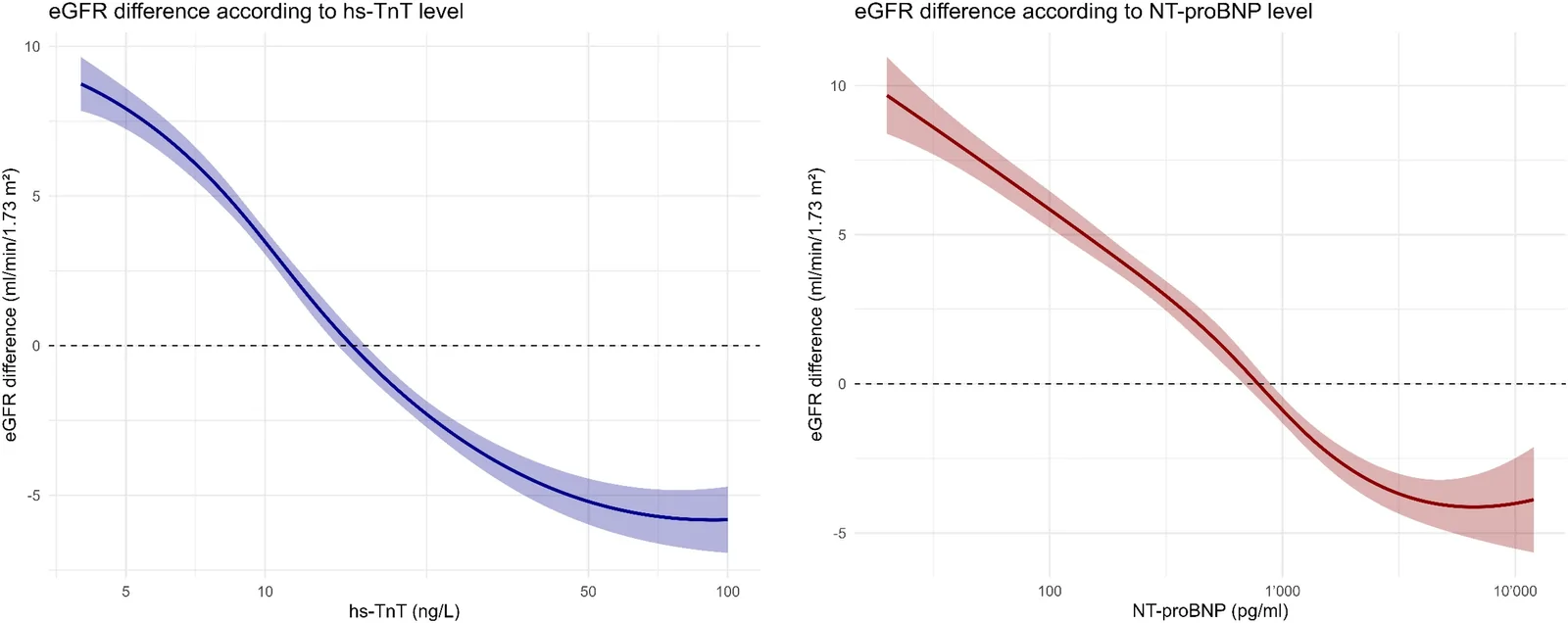
Relation of estimated glomerular filtration rate difference with high-sensitivity troponin T (left panel) and NT-proBNP (right panel).

### eGFR difference and adverse outcomes

During a median follow-up of 8 years, 985 MACE and 831 HF events occurred. The annual incidence rates increased from 1.69 to 3.90 to 6.78 for MACE and from 1.24 to 3.48 to 6.07 for HF events across >10, −10 to 10, and <−10 mL/min/1.73 m^2^ eGFRdiff groups (*Figure 4*). Cumulative incidence curves for the two main outcomes are shown in *Figure 5*. The respective curves of the <−10 mL/min/1.73 m^2^ groups increased substantially more compared to the other two eGFRdiff groups.

**Figure 4:**
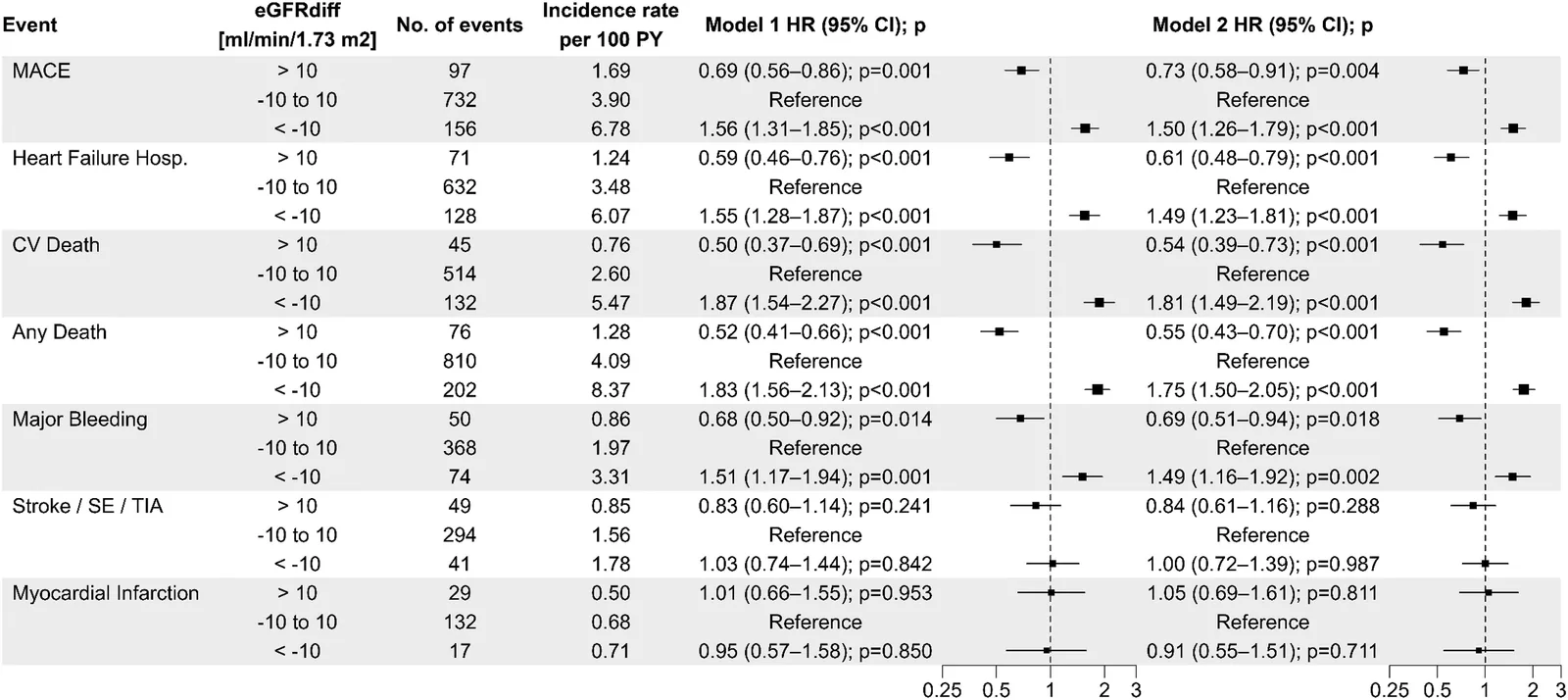
Association of estimated glomerular filtration rate difference with cardiovascular events. Model 1 was adjusted for age and sex. Model 2 was adjusted for age, sex, diabetes, arterial blood pressure, history of stroke/transient ischaemic attack, and smoking status. eGFRdiff = eGFRcrea-cys – eGFRcrea.

**Figure 5:**
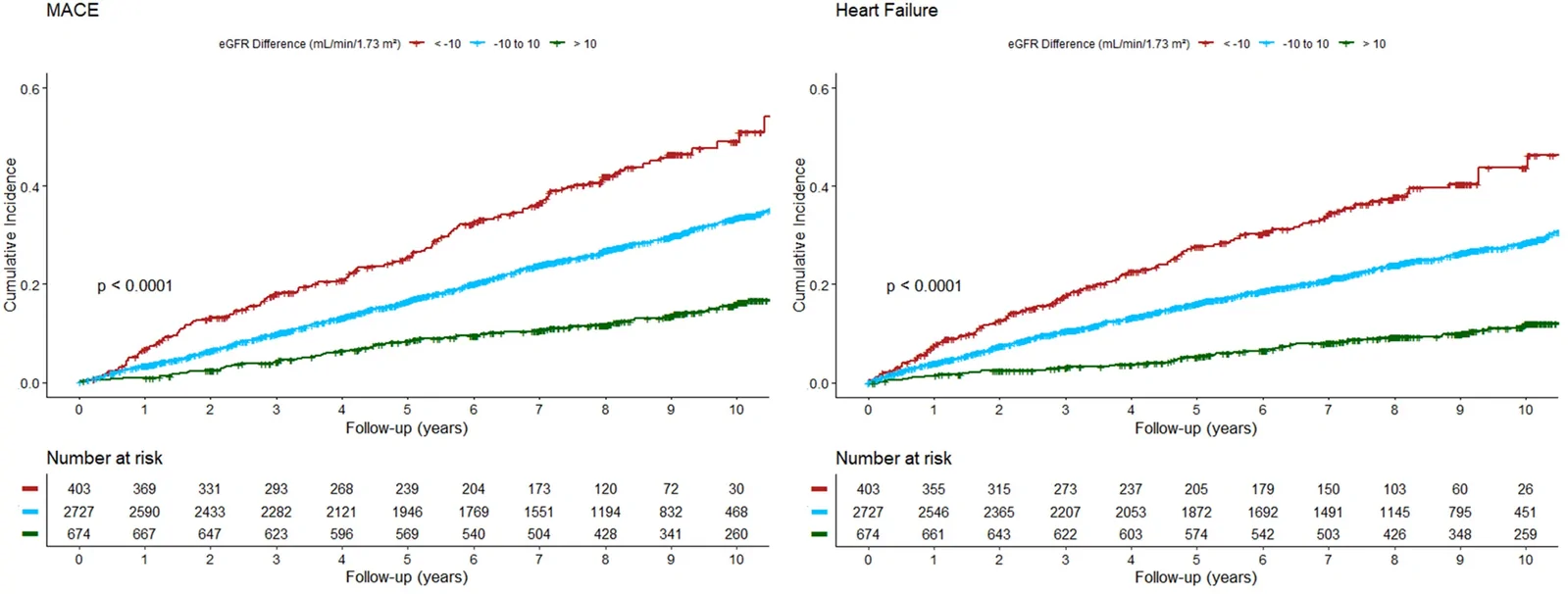
Incidence curves for major cardiovascular events (left panel) and first heart failure hospitalization (right panel) according to estimated glomerular filtration rate difference groups. eGFRdiff = eGFRcrea-cys – eGFRcrea.

In Cox-proportional hazards regression models, eGFRdiff categories were significantly associated with both primary outcomes (*Figure 4*). Using the eGFRdiff group −10 to 10 mL/min/1.73 m^2^ as the reference, patients with an eGFRdiff <−10 mL/min/1.73 m^2^ had an adjusted hazard ratio (95% confidence interval) of 1.50 (1.26; 1.79; *P* < 0.001) for MACE and of 1.49 (1.23; 1.81, *P* < 0.001) for HF hospitalizations. Similarly, eGFRdiff was significantly associated with both outcomes when used as a continuous variable (*Figure 6*). The results were consistent for CV death, all-cause death, and major bleeding, but not for stroke or systemic embolism and myocardial infarction (*Figure 4*). In sensitivity and specificity analyses, the <−10 mL/min/1.73 m^2^ cut-off had a very high sensitivity to identify high-risk patients and the >10 mL/min/1.73 m^2^ cut-off had a very high specificity (see Supplementary material online, *Table S2*). The calculated optimal cut-off using the Youden index was around 0 for different follow-up periods (see Supplementary material online, *Table S3***)**.

**Figure 6:**
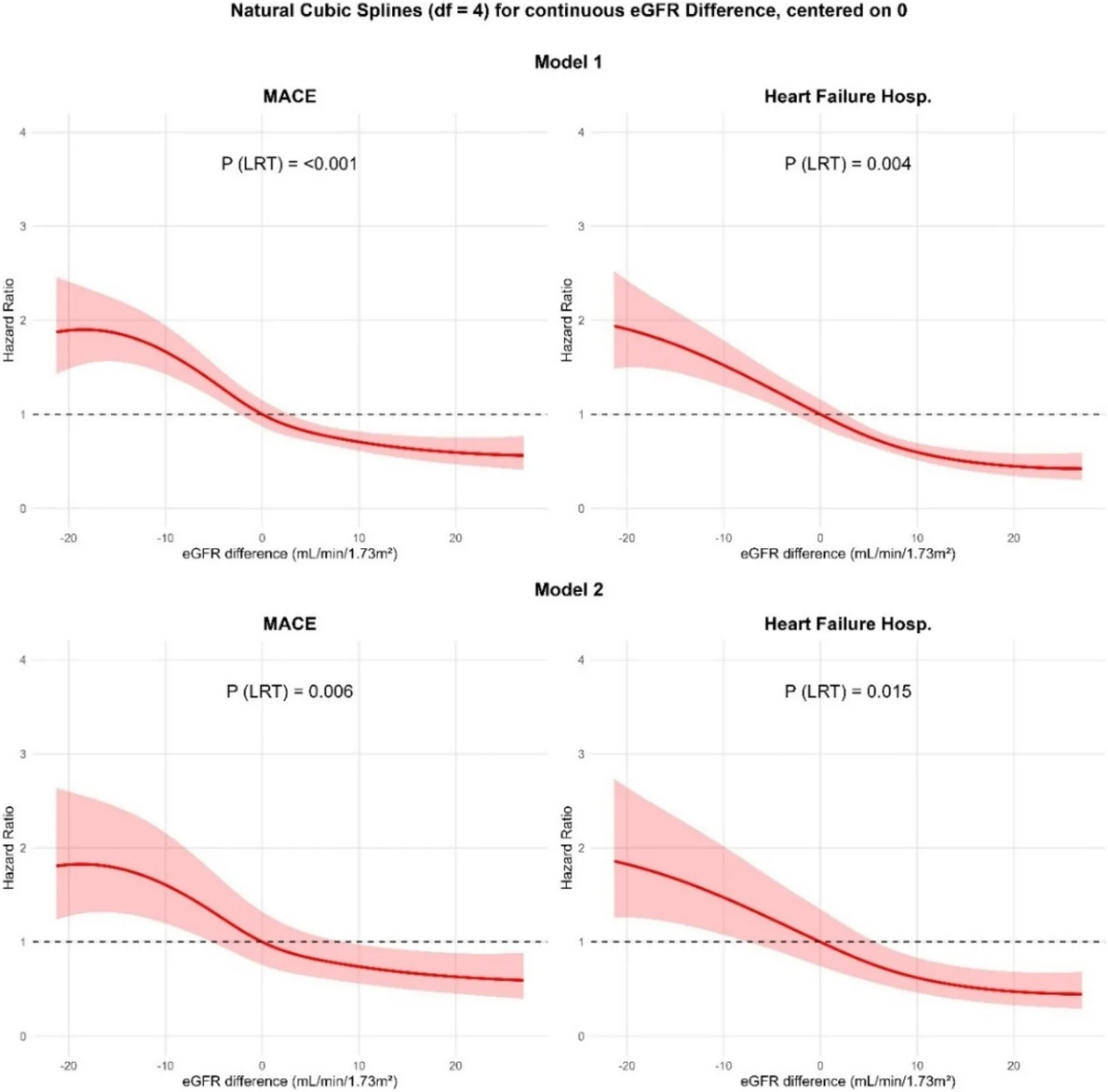
Association of estimated glomerular filtration rate difference with cardiovascular events. Model 1 was adjusted for age and sex. Model 2 was adjusted for age, sex, diabetes, arterial blood pressure, history of stroke/TIA, and smoking status. eGFRdiff = eGFRcrea-cys – eGFRcrea.

Time-dependent AUC (95% CI) at 10 years for MACE and HF hospitalization were highest for eGFRcrea-cys (0.772 and 0.803, respectively) followed by eGFRdiff (0.742 and 0.756, respectively) (see Supplementary material online, *Table S4* and Supplementary material online, *Figure S4*). Adding eGFRdiff to eGFRcrea and eGFRcrea-cys increased all AUCs, though the increase was smaller for models with eGFRcrea-cys (see Supplementary material online, *Table S5*).

## Discussion

In this prospective cohort of patients with AF, about 30% had a clinically relevant difference exceeding 10 mL/min/1.73 m^2^ between creatinine- and cystatin C-based eGFR (eGFRdiff). The eGFRdiff was strongly associated with elevated cardiac biomarkers and adverse outcomes. Patients with higher eGFRcrea compared to eGFRcrea-cys showed higher levels of hs-TnT and NT-proBNP, and an increased risk for MACE, HF events, and prevalent brain lesions. These findings suggest that eGFRdiff may identify a high-risk subset of AF patients and may have potential implications for pharmacologic management, particularly for renal function-guided drug dosing.

A considerable proportion of AF patients had a clinically relevant eGFRdiff of >10 mL/min/1.73 m^2^, raising concerns regarding inappropriate dosing of DOAC. These findings raise the question, which eGFR estimation method should be used in clinical practice for drug dosing in AF patients. In the landmark DOAC trials ROCKET-AF and ENGAGE AF-TIMI 48, creatinine clearance calculated by the Cockcroft–Gault equation was used for dose adjustment, while creatinine was directly used in ARISTOTLE and no dose adjustment criteria were applied in RE-LY.^20–23^ In clinical practice, the latest CKD-EPI equation for eGFRcrea is most commonly used for dose adjustment. While there is little difference between different iterations of the CKD-EPI equation,^24^ the correlation between creatinine clearance, eGFRcrea, and eGFRcrea-cys varies substantially.^25,26^ In a post-hoc analysis of ARISTOTLE, cystatin c-based eGFR was most robust for predicting death and bleeding.^25^ Our prior analysis of this cohort supports that observation: cystatin c-based eGFR was associated with both ischaemic and bleeding outcomes, while creatinine-based eGFR was only associated with ischaemic outcomes.^4^ These findings underscore the need for harmonization and prospective validation, ideally within large pooled datasets such as the COMBINE-AF dataset that includes all landmark DOAC trials.^27^

A negative eGFRdiff was most pronounced among patients with higher disease burden, as reflected by higher levels of hs-TnT and NT-proBNP. Creatinine is primarily released by muscles, partially actively secreted by the proximal tubule and excreted by the kidneys and to a lesser extent by the gastro-intestinal system.^28^ As patients with more advanced HF have less muscle mass in addition to potentially increased tubular creatinine secretion due to neurohumoral activation, creatinine levels might be less accurate for estimating kidney function. Moreover, a more negative eGFRdiff might also reflect greater congestion by more severe HF and worse kidney function, rather than intrinsic glomerular damage.^9^ Conversely, cystatin C is continually synthesized in all nucleated cells, not actively secreted and, due to its low molecular weight, almost completely filtered in the glomerulus and catabolized in the proximal tubule.^28,29^ This leads to a higher stability in serum concentration, a reduced sensitivity to confounding factors and a more accurate estimation of kidney function.^30,31^ These biochemical characteristics translate into more consistent associations of cystatin C-based eGFR with ischaemic and bleeding events, compared to creatinine-based eGFR.^4^

In patients with AF, our findings add to the role of renal function as part of a multidomain risk profile, rather than an isolated marker, within the CV-kidney-metabolic (CKM) framework.^32,33^ Patients with AF have a particularly high burden of CKM domains, that were associated both with adverse outcomes and treatment decisions.^32^ Specifically, AF patients with multiple CKM domains, like systolic blood pressure, HbA1c, and triglyceride levels, have highly complex clinical profiles, that influence pharmacological and lifestyle interventions.^33^ Pathophysiologically, cardiorenal interactions lead to sympathetic nervous system activation with vasoconstriction, an activated renin–angiotensin–aldosterone system, chronic inflammation with oxidative stress and endothelial dysfunction. These mechanisms further impair the glomerular filtration by damaging the renal microvasculature and fibrotic remodelling.^34,35^

Patients with an eGFRdiff <−10 mL/min/1.73 m^2^ had a four-fold higher risk of MACE, nearly five-fold increased HF hospitalization risk, and a greater burden of ischaemic brain lesions, compared to patients with an eGFRdiff >10 mL/min/1.73 m^2^. Specifically, both overt and covert ischaemic brain lesions were associated with cognitive impairment in prior analyses of our cohort.^16,17^ Similar observations of clinical outcomes have been shown in patients with CKD and HF.^8–10^ In patients with CKD a negative eGFRdiff associates with worse outcomes in 5000 In the Chronic Renal Insufficiency Cohort study and in a meta-analysis of more than 800 000 patients.^8,10^ Moreover, a change in eGFRdiff over time was associated with an increased risk for mortality in CKD patients.^8^ In the PARADIGM-HF study, a significant eGFRdiff was linked to biomarker profiles of advanced HF and poor prognosis.^9^ Our study extends these findings to the growing AF population, where accurate renal function assessment is essential to guide drug dosing. The clinical impact of potentially inappropriate drug dosing needs to be further investigated in other large AF patient populations. The translation of our current findings into clinical practice is currently limited by the necessity of simultaneous availability of both biomarkers.

### Strength and limitations

Strengths of our study include the large, prospectively enrolled, well-defined population with standardized outcome adjudication by an independent clinical committee. The availability of biomarker and MRI data enhances mechanistic insights. The following limitations should be acknowledged. Biomarker measurements were limited to baseline precluding longitudinal assessment. Brain MRI was only available in the Swiss-AF cohort with potential selection bias. Directly measured GFR was not available, preventing direct comparison to a reference standard. While the results are generalizable to patients with AF in Switzerland, caution is warranted when extrapolating to other geographical or disease populations. The observational design limits causal inference, and residual confounding, by for example comorbidity burden or unmeasured frailty indices, cannot be excluded. Reverse causation may also be present, whereby patients with more advanced CV disease exhibit both altered biomarker profiles and greater eGFR discordance.

## Conclusion

A substantial proportion of patients with AF had clinically relevant discrepancies between creatinine- and cystatin C-based eGFR estimates. A negative eGFRdiff was associated with elevated cardiac biomarkers, adverse CV outcomes, and increased burden of brain lesions. eGFRdiff may serve a marker of cardiorenal and cerebrovascular risk in AF and warrants further investigation to clarify its role in guiding treatment decisions.

## Supplementary Material

oeag145_Supplementary_Data

## Data Availability

The data that support the findings of this study are not openly available due to reasons of sensitivity and are available from the corresponding author upon reasonable request.
