# Supplementary material for: Impact of differences in kidney function estimation by creatinine compared to cystatin C on clinical outcomes in patients with atrial fibrillation
Source: Eur Heart J Open. 2026 Sep 22;6(5):oeag145. doi: 10.1093/ehjopen/oeag145 (PMC13643174; doi:10.1093/ehjopen/oeag145)
Supplement: Supplementary file 1 [file oeag145_supplementary_data.docx]

**SUPPLEMENTARY MATERIAL**

**Impact of Differences in Kidney Function Estimation by Creatinine compared to Cystatin C** **on Clinical Outcomes in Patients with Atrial Fibrillation**

**Index**

Swiss-AF investigators	3

Outcome definitions	6

Supplementary Table S1	8

Supplementary Table S2	10

Supplementary Table S3	11

Supplementary Table S4	12

Supplementary Table S5	13

Supplementary Figure S1	14

Supplementary Figure S2	15

Supplementary Figure S3	16

Supplementary Figure S3	17

**Swiss-AF investigators**

University Hospital Basel and Basel University: Stefanie Aeschbacher, Steffen Blum, Leo H. Bonati, Désirée Carmine, David Conen, Ludvig Dahlheim, Corinne Girroy, Andreas Gasser, Elisa Hennings, Ruben Kerler, Philipp Krisai, Michael Kühne, Vincent Meier, Christine Meyer-Zürn, Pascal B. Meyre, Andreas U. Monsch, Christian Müller, Stefan Osswald, Rebecca E. Paladini, Raffaele Peter, Adrian Schweigler, Christian Sticherling, Gian Völlmin.

Principal Investigator: Stefan Osswald; Local Principal Investigator: Michael Kühne

University Hospital Bern: Faculty: Drahomir Aujesky, Urs Fischer, Juerg Fuhrer, Laurent Roten, Simon Jung, Heinrich Mattle; Research fellows: Seraina Netzer, Luise Adam, Carole Elodie Aubert, Martin Feller, Axel Loewe, Elisavet Moutzouri, Claudio Schneider; Study nurses: Tanja Flückiger, Cindy Groen, Lukas Ehrsam, Sven Hellrigl, Alexandra Nuoffer, Damiana Rakovic, Nathalie Schwab, Rylana Wenger, Tu Hanh Zarrabi Saffari. Local Principal Investigators: Nicolas Rodondi, Tobias Reichlin

Stadtspital Triemli Zurich: Alain Bernheim, Roger Dillier, Michèle Deubelbeiss, Franz Eberli, Christine Franzini, Isabel Juchli, Claudia Liedtke, Samira Murugiah, Jacqueline Nadler, Thayze Obst, Jasmin Roth, Fiona Schlomowitsch, Xiaoye Schneider, Peter Sporns, Katrin Studerus, Noreen Tynan, Dominik Weishaupt. Local Principal Investigator: Andreas Müller

Kantonspital Baden: Corinne Friedli, Silke Kuest, Karin Scheuch, Denise Hischier, Nicole Bonetti, Alexandra Grau, Jonas Villinger, Eva Laube, Marcel Frick, Stefanie Leuenberger, Franziska Rutz. Local Principal Investigator: Jürg-Hans Beer

Cardiocentro Lugano: Angelo Auricchio, Adriana Anesini, Cristina Camporini, Maria Luce Caputo, Rebecca Peronaci, Francois Regoli, Martina Ronchi. Local Principal Investigator: Giulio Conte

Kantonsspital St. Gallen: Roman Brenner, David Altmann, Karin Fink, Michaela Gemperle, Jessica Meier. Local Principal Investigator: Peter Ammann

Hôpital Cantonal Fribourg: Mathieu Firmann, Sandrine Foucras, Martine Rime. Local Principal Investigator: Michael Kühne

Luzerner Kantonsspital: Benjamin Berte, Kathrin Bühler, Amir Jadidi, Virgina Justi, Frauke Kellner-Weldon, Richard Kobza, Melanie Koch, Brigitta Mehmann, Sonja Meier, Myriam Roth, Andrea Ruckli-Kaeppeli, Ian Russi, Kai Schmidt, Mabelle Young. Local Principal Investigator: Michael Kühne

Ente Ospedaliero Cantonale Lugano: Elia Rigamonti, Carlo Cereda, Alessandro Cianfoni, Maria Luisa De Perna, Jane Frangi-Kultalahti, Patrizia Assunta Mayer Melchiorre, Anica Pin, Serena Staglianò, Tatiana Terrot, Luisa Vicari. Local Principal Investigator: Giorgio Moschovitis.

University Hospital Geneva: Georg Ehret, Hervé Gallet, Elise Guillermet, Francois Lazeyras, Karl-Olof Lovblad, Patrick Perret, Philippe Tavel, Cheryl Teres. Local Principal Investigator: Dipen Shah

University Hospital Lausanne: Nathalie Lauriers, Marie Méan, Alessandra Pia Porretta, Sandrine Salzmann, Jürg Schläpfer. Local Principal Investigator: Michael Kühne

Bürgerspital Solothurn: Andrea Grêt, Jan Novak, Sandra Vitelli. Local Principal Investigator: Frank-Peter Stephan

Ente Ospedaliero Cantonale Bellinzona: Jane Frangi-Kultalahti, Augusto Gallino, Luisa Vicari. Local Principal Investigator: Marcello Di Valentino

University of Zurich/University Hospital Zurich: Helena Aebersold, Fabienne Foster, Matthias Schwenkglenks.

Medical Image Analysis Center AG Basel: Marco Düring, Tim Sinnecker, Anna Altermatt, Michael Amann, Petra Huber, Manuel Hürbin, Esther Ruberte, Alain Thöni, Jens Würfel, Vanessa Zuber.

Department Clinical Research: Michael Coslovsky, Pia Neuschwander, Patrick Simon,

Schiller AG Baar: Ramun Schmid, Christian Baumann

**Outcome definitions**

**Stroke**

Stroke was defined as an acute focal neurologic deficit of vascular origin, confirmed by imaging (computed tomography or MRI) or autopsy.

**Systemic embolism**

A systemic arterial embolism was considered to have occurred where there was clear evidence of abrupt occlusion of a systemic artery consistent with an embolic event. Pulmonary embolism or deep vein thrombosis were not included.

Two criteria were required for event to be defined as Systemic Arterial Embolism for the study outcome:

1. Clinical signs and symptoms consistent with embolic arterial occlusion

2. At least one of the following objective findings:

o	Surgical report indicating evidence of arterial embolism.

o	Pathological specimens related to embolism removal.

o	Imaging evidence consistent with arterial embolism.

o	Autopsy reports.

**Death**

Deaths were classified as either cardiovascular or non-cardiovascular. All deaths were assumed to be cardiovascular in nature unless a non-cardiovascular cause could have been clearly provided. These included cardiac deaths (e.g. cardiogenic shock, arrhythmia/sudden death, cardiac rupture) and other vascular deaths (e.g., stroke, pulmonary embolism, ruptured aortic aneurysm or dissection). All hemorrhagic deaths were not classified as cardiovascular deaths.

**Major bleeding**

Major bleeding was defined according to the criteria of the International Society on Thrombosis and Haemostasis, as at least one of the following:

-	Fatal bleeding.

-	Symptomatic bleeding in a critical area or organ. (Intracranial, Intraspinal, Intraocular, Retroperitoneal, Intra-articular, Pericardial, Intramuscular - with compartment syndrome)

-	Bleeding causing a fall in hemoglobin level of 2 g/dL (20 g/L) within 7 days or leading to transfusion of two or more units of whole blood or red cells.

**Myocardial infarction**

Myocardial infarction was defined according to the universal definition of MI as rise and/or fall of cardiac troponin with at least one value above the 99th percentile of the upper reference limit in a clinical setting consistent with myocardial ischemia, and with at least one of the following:

-	Symptoms of ischemia.

-	New ST elevation at the J point in two contiguous leads >0.1 mV except for V2- V3. For leads V2-V3 the following cut points apply: ≥0.2 mV in men ≥40 years, ≥0.25 mV in men <40 years and ≥0.15 mV in women on ECG.

-	New horizontal or down-sloping ST depression ≥0.05 mV in two contiguous leads and/or T inversion ≥0.1 mV in two contiguous leads with prominent R wave or R/S ratio >1.

-	New left bundle brunch block on ECG.

-	Development of pathological Q waves on ECG.

-	Imaging evidence of new loss of viable myocardium or new regional wall motion.

-	Identification of an intracoronary thrombus by angiography or autopsy.

**Hospitalization for heart failure**

Hospitalization for acute heart failure was defined as: any hospitalization for acute heart failure that was associated with at least one overnight stay. If it was not clear whether the reason for a patient’s hospitalization was acute heart failure or not, this event/incidence was in doubt classified as acute heart failure. The following signs were used as an indication for heart failure: leg swelling/leg edema, distension of the neck veins, positive hepato-jugular reflux, rales and 3rd heart sound

**Supplementary Table S1**	Baseline characteristics stratified by study cohort.

| Variable | Beat-AF | Swiss-AF | p-value |
| --- | --- | --- | --- |
| Age (years) | 69 (±12) | 73 (±8) | <0.001 |
| Sex (male) | 1,040 (71%) | 1,703 (73%) | 0.100 |
| BMI (kg/m²) | 27.1 (±4.7) | 27.7 (±4.8) | <0.001 |
| Systolic blood pressure (mmHg) | 134 (±18) | 134 (±19) | 0.859 |
| Diastolic blood pressure (mmHg) | 78 (±12) | 77 (±12) | 0.033 |
| Heart rate (bpm) | 67 (58, 79) | 66 (59, 76) | 0.030 |
| Smoking status |  |  | 0.064 |
| Active | 130 (8.9%) | 168 (7.2%) |  |
| non-active | 1,335 (91%) | 2,161 (93%) |  |
| CHA₂DS₂-VASc Score | 2.79 (±1.73) | 3.47 (±1.70) | <0.001 |
| Atrial fibrillation type |  |  | <0.001 |
| paroxysmal | 824 (56%) | 1,038 (45%) |  |
| non-paroxysmal | 646 (44%) | 1,293 (55%) |  |
| Rhythm at baseline |  |  | <0.001 |
| SR | 800 (55%) | 1,071 (46%) |  |
| AF | 589 (40%) | 877 (38%) |  |
| other | 69 (4.7%) | 372 (16%) |  |
| Prior Pulmonary Vein Isolation | 336 (23%) | 471 (20%) | 0.053 |
| Coronary Artery Disease | 309 (21%) | 708 (30%) | <0.001 |
| Prior Stroke/TIA | 189 (13%) | 458 (20%) | <0.001 |
| Hypertension | 999 (68%) | 1,627 (70%) | 0.209 |
| Heart Failure | 298 (20%) | 605 (26%) | <0.001 |
| Diabetes | 205 (14%) | 409 (18%) | 0.003 |
| Prior Myocardial Infarction | 185 (13%) | 377 (16%) | 0.002 |
| Prior major and clinically relevant non-major bleed | 141 (9.6%) | 360 (15%) | <0.001 |
| Prior Systemic Embolism | 67 (4.6%) | 123 (5.3%) | 0.317 |
| Creatinine [mg/dl] | 1.04 (0.89, 1.23) | 1.13 (0.97, 1.36) | <0.001 |
| Cystatine C [mg/dl] | 0.99 (0.84, 1.23) | 1.20 (1.01, 1.49) | <0.001 |
| eGFRcrea [mL/min/1.73 m²] | 73.19 (58.18, 86.92) | 63.26 (50.21, 76.78) | <0.001 |
| eGFRcrea-cys [mL/min/1.73 m²] | 77.68 (59.42, 94.32) | 62.98 (47.68, 77.34) | <0.001 |
| eGFR-Differenz [mL/min/1.73 m²] | 3.78 (-2.46, 10.77) | -1.05 (-6.16, 4.89) | <0.001 |
| High-sensitivity Troponin T [ng/L] | 10.72 (6.91, 17.59) | 14.46 (9.48, 23.89) | <0.001 |
| NT-proBNP [ng/L] | 448 (134, 1,175) | 646 (218, 1,465) | <0.001 |
| Beta Blockers | 976 (66%) | 1,641 (70%) | 0.007 |
| ACE Inhibitors | 355 (24%) | 744 (32%) | <0.001 |
| Calcium Antagonist | 244 (17%) | 515 (22%) | <0.001 |
| Angiotensin Receptor Blockers | 407 (28%) | 746 (32%) | 0.004 |
| Digoxin | 57 (3.9%) | 101 (4.3%) | 0.485 |
| Diuretics | 499 (34%) | 1,098 (47%) | <0.001 |
| NOAK | 154 (10%) | 1,188 (51%) | <0.001 |
| Vitamin K Antagonists | 939 (64%) | 917 (39%) | <0.001 |
| Antiplatelet Therapy | 360 (25%) | 460 (20%) | <0.001 |
| Antiarrhythmics, Class Ic and III | 384 (26%) | 507 (22%) | 0.002 |

**Supplementary Table S2** Sensitivity and specificity for pre-specified eGFRdiff cut-offs.

| **Endpoint** | **eGFRdiff threshold** | **Sensitivity (%)** | **Specificity (%)** |
| --- | --- | --- | --- |
| **MACE** | < −10 | 94.0 | 22.4 |
|  | > +10 | 25.9 | 93.1 |
| **Heart Failure Hospitalisation** | < −10 | 93.2 | 22.6 |
|  | > +10 | 24.3 | 93.5 |

**Supplementary Table S3** Optimal eGFRdiff cut-off by Youden-index for different follow-up periods.

| **Endpoint** | **Time (years)** | **Optimal cutoff (eGFRdiff)** | **Sensitivity (%)** | **Specificity (%)** | **Youden index** |
| --- | --- | --- | --- | --- | --- |
| MACE | 3 | 1.1 | 72.7 | 55.6 | 0.283 |
| Heart Failure Hosp. | 3 | 1.2 | 70.9 | 56.7 | 0.276 |
| MACE | 5 | 1.0 | 69.8 | 57.6 | 0.274 |
| Heart Failure Hosp. | 5 | 1.2 | 72.4 | 60.1 | 0.325 |
| MACE | 10 | -0.8 | 72.5 | 67.2 | 0.396 |
| Heart Failure Hosp. | 10 | -0.9 | 73.3 | 68.8 | 0.420 |

**Supplementary Table S4**	10-year time-dependent AUC of ROC for eGFRdiff vs. eGFRcrea vs eGFRcrea-cysc.

| **Endpoint** | **Marker** | **AUC (95% CI)** |
| --- | --- | --- |
| **MACE** | eGFRdiff | 0.742 (0.717-0.766) |
|  | eGFRcrea | 0.706 (0.681-0.731) |
|  | eGFRcrea-cys | 0.772 (0.748-0.795) |
| **Heart Failure Hosp.** | eGFRdiff | 0.756 (0.730-0.782) |
|  | eGFRcrea | 0.736 (0.710-0.762) |
|  | eGFRcrea-cys | 0.803 (0.780-0.827) |

Model 2 includes age, sex, diabetes, arterial blood pressure, history of stroke/TIA, and smoking status.

**Supplementary Table S5**	Increase in AUC by adding eGFRdiff to either eGFRcrea or eGFRcrea-cys.

| **Endpoint** | **Base model** | **C-statistic (base model)** | **C-statistic (+ eGFRdiff)** | **ΔC** | **95% CI (ΔC)** | **p-value** |
| --- | --- | --- | --- | --- | --- | --- |
| **MACE** | Model 2 + eGFRcrea | 0.715 (0.698–0.731) | 0.729 (0.713–0.745) | 0.0142 | 0.0074 to 0.0210 | <0.001 |
|  | Model 2 + eGFRcrea-cys | 0.723 (0.707–0.739) | 0.729 (0.713–0.745) | 0.0058 | 0.0016 to 0.0100 | 0.006 |
| **Heart Failure Hospitalisation** | Model 2 + eGFRcrea | 0.743 (0.727–0.760) | 0.763 (0.747–0.779) | 0.0197 | 0.0114 to 0.0280 | <0.001 |
|  | Model 2 + eGFRcrea-cys | 0.757 (0.741–0.773) | 0.763 (0.747–0.779) | 0.0060 | 0.0017 to 0.0102 | 0.006 |

Model 2 includes age, sex, diabetes, arterial blood pressure, history of stroke/TIA, and smoking status.

**Supplementary Figure S1** Study flow.

**Supplementary Figure S2**	Relation of creatinine and cystatin C with high-sensitivity Troponin T and NT-proBNP.

**Supplementary Figure S3**	Relation of creatinine and cystatin C with NT-proBNP.

**Supplementary Figure S4**	Time-dependent AUC at 10 years using IPCW weighting. 95% CI from I	nfluence function. N = 3804 patients; MACE events = 985; HF events = 831..
